# Antimicrobial Resistance in Commensal Flora of Pig Farmers

**DOI:** 10.3201/eid1005.030735

**Published:** 2004-05

**Authors:** Hélène Aubry-Damon, Karine Grenet, Penda Sall-Ndiaye, Didier Che, Eugenio Cordeiro, Marie-Elisabeth Bougnoux, Emma Rigaud, Yann Le Strat, Véronique Lemanissier, Laurence Armand-Lefèvre, Didier Delzescaux, Jean-Claude Desenclos, Michel Liénard, Antoine Andremont

**Affiliations:** * National Institute for Public Health, Saint-Maurice, France; † Bichat Hospital, Assistance Publique, Paris, France; ‡ National Medical Insurance System for Agriculture, Bagnolet, France; § National Federation of Cattle and Pig Raisers, Paris, France; ¶ Hôpital Ambroise Paré, Paris, France

**Keywords:** Drug resistance, bacterial

## Abstract

We assessed the quantitative contribution of pig farming to antimicrobial resistance in the commensal flora of pig farmers by comparing 113 healthy pig farmers from the major French porcine production areas to 113 nonfarmers, each matched for sex, age, and county of residence. All reported that they had not taken antiimicrobial agents within the previous month. Throat, nasal, and fecal swabs were screened for resistant microorganisms on agar containing selected antimicrobial agents. Nasopharyngeal carriage of *Staphylococcus aureus* was significantly more frequent in pig farmers, as was macrolide resistance of *S. aureus* from carriers. Nongroupable streptococci from the throat were more resistant to the penicillins in pig farmers. The intestinal isolation of enterococci resistant to erythromycin or vancomycin was not significantly higher in pig farmers in contrast to that of enterobacteria resistant to nalidixic acid, chloramphenicol, tetracycline, and streptomycin. Prevalence of resistance in predominant fecal enterobacteria was also significantly higher in pig farmers for cotrimoxazole, tetracycline, streptomycin, and nalidixic acid. We determined a significant association between pig farming and isolation of resistant commensal bacteria.

Higher prevalence of antimicrobial-resistant bacteria in commensal flora contributes to the general increase and dissemination of bacterial resistance worldwide (*1*,*2*) and can be a source of resistance genes for respiratory pathogens such as *Streptococcus pneumoniae* (*3*) and intestinal pathogens such as *Shigella* (*4*) or *Salmonella* (*5*,*6*). Antimicrobial treatments are major factors for selection of resistance in the commensal flora of humans (*7*). Industrial animal farming is also associated with large-scale antimicrobial use (*8*), which leads to a high level of colonization of animals with antimicrobial-resistant bacteria that can then contaminate the food and in turn, humans (*9*,*10*). Farmers are more likely to acquire enteric antimicrobial-resistant bacteria from food-producing animals, even if not treated with antimicrobial agents, themselves (*11*–*14*). However, this link has never been quantitatively assessed. Antimicrobial resistance in nasal and pharyngeal commensal strains might possibly be affected in the same manner, and this hypothesis has also not been investigated. We thus designed an exposed-nonexposed epidemiologic study to determine the association between contact with animals in pig-raising farms and isolation of antimicrobial-resistant nasal, pharyngeal, and intestinal commensal microorganisms.

## Methods

### Participants

The study population was composed of members of the Mutualité Sociale Agricole (MSA), a health insurance system for workers in agriculture and related services. We identified pig farmers as an exposed group and nonfarmers (such as those working at banks or in insurance services) as a nonexposed group. The sample size was calculated according to results on the prevalence of antimicrobial resistance in the fecal flora of French residents (*15*) to ensure that, for most markers measured, detection of a 10% difference in the exposed group would be found with a power of 80% and an α risk of 5%. Pig farmers were chosen among those working in large, exclusively pig farms (>84 pigs) and contacted during the yearly MSA preventive medicine visits to obtain permission for participation. One pig farmer per farm was randomly selected to fill a panel of 20 in each of the seven major French porcine production areas.

One nonfarmer control, matched for sex, age, and county of residence, was selected for each pig farmer and approached similarly. Nonfarmers were not living or working on a farm, in a slaughterhouse, or in the pharmaceutical industry and were not living with someone who worked on a farm.

Persons included in the study were judged healthy by physical examination, had no gastrointestinal symptoms or throat pain at inclusion, and reported that they had not been hospitalized or taken antimicrobial agents within the previous month. All study participants were enrolled within 3 months. Study participants’ antimicrobial use in the 6 months preceding the study was retrospectively estimated from the MSA reimbursement database and converted to defined daily doses, as described (*16*). In cases in which methicillin-resistant *Staphylococcus aureus* (MRSA) was isolated, participants were further interviewed for hospitalization and contacts with hospitalized patients and healthcare workers during the previous year, as described (*17*). Occurrence and type of contact with pigs and contact precautions used in farms were documented in pig farmers with a standardized questionnaire. This study was performed in agreement with legal and ethical French regulatory procedures.

### Specimens Obtained

Study participants were asked to bring fresh stool samples in sterile, closed cups. A sterile cotton swab was immersed in the sample. No procedure was implemented to ensure that participants brought their own stool specimens. They likely did, however, since participants were contacted during the yearly MSA preventive medicine visits by the practitioner with whom they had an established confidential relationship. Nasal swabs were inserted (1 cm) successively in both nares and rotated three times for 10 to 15 s. Pharyngeal samples were obtained by firmly pressing a swab over the tonsils and the posterior pharyngeal wall, and avoiding touching the jaws, teeth, or gingival when withdrawing the swab. All swabs were extemporaneously squeezed in sterile brain-heart infusion broth (BioMérieux, Marcy-l’Etoile, France) with 10% glycerol, immerged in liquid nitrogen within 6 hours, and stored at –80°C until processing.

### Detection of Microbial Isolates

One hundred microliter–aliquots of all broth samples were plated as follows. For nasal samples, isolation of *S. aureus* was performed on Chapman agar (BioMérieux). Antimicrobial susceptibility of one isolate per participant was determined by using the disk diffusion technique (*18*).

For the pharyngeal samples, isolation of *Streptococcus pneumoniae* and β-hemolytic streptococci was performed on 5% sheep blood Columbia agar; isolation of *Haemophilus influenzae* was performed on chocolate agar*, Staphylococcus aureus* on Chapman agar, and yeast on Chromagar (all BioMérieux). Isolation of antimicrobial-resistant nongroupable streptococci was performed on 5% sheep blood Columbia agar supplemented with nalidixic acid and colistin. Antimicrobial-resistant nongroupable streptococci were detected on the same medium, supplemented with ampicillin (4 mg/L) or erythromycin (1 mg/L). For feces, aliquots were plated on Chromagar, Cetrimide (Bio-Mérieux), and Chapman agar for detection of yeasts, *Pseudomonas aeruginosa,* and *S. aureus*, respectively. Detection of enterococci of any resistance phenotype and of those resistant to erythromycin was performed on Bile-Esculin-agar (BEA) (BioMérieux) free of antimicrobial agents or supplemented with 5 mg erythromycin/L, respectively. Detection of vancomycin-resistant enterococci (VRE) was performed on BEA supplemented with 10 mg vancomycin/L after an enrichment step of 18 hours in broth containing 1 mg vancomycin/L, as described (*19*,*20*). The mechanism of vancomycin resistance was determined by polymerase chain reaction analysis, as described (*21*). Carriage of resistant enterobacteria was detected by using two separate procedures, as described (*22*), with modifications. In the first, designed to explore the subdominant flora, 0.1 mL of broth was plated on Drigalski agar supplemented with ampicillin (10 mg/L), ceftazidime (2 mg/L), streptomycin (20 mg/L), kanamycin (20 mg/L), chloramphenicol (20 mg/L), tetracycline (10 mg/L), or nalidixic acid (50 mg/L), as described (*15*). *Escherichia coli* of known susceptibility were used as the control. One of 10 positive plates was selected for quality control, and one colony was selected for antimicrobial susceptibility testing. A study participant was defined as colonized in the subdominant fecal flora with enterobacteria resistant to a given antimicrobial agent when at least one colony grew from the plate containing the corresponding antimicrobial agent.

In the second procedure, designed to explore the predominant fecal flora, Drigalski agar plates without antimicrobial agents were spread with 0.1 mL of broth culture. Five colonies were randomly selected. Those identified as *E. coli* were tested for antimicrobial susceptibility. A study participant was defined as colonized in the predominant flora by *E. coli* resistant to a given antimicrobial agent when at least one resistant strain was recovered from the feces by using this second procedure.

### Statistical Analysis

The prescribed defined daily doses of an antimicrobial agent and the number of participants who had ordered antimicrobial agents within the previous 6 months were compared between pig farmers and nonfarmers by using the Student *t* test for matched data. Differences between groups for carriage of nasal, pharyngeal, and fecal microbial species were analyzed by calculating matched prevalence ratios (PR) (*23*). For comparing antimicrobial-resistant phenotypes of *S. aureus*, nongroupable streptococci, *E. coli*, enterococci, and enterobacteria from pig farmers and nonfarmer carriers, nonmatched PR were used, since these comparisons were performed on subgroups composed of only the carriers of the species among the resistant clones for which we looked. (For instance, rates of carriage of resistant enterobacteria were composed from subgroups of those actually carrying enterobacteria.) Because this analysis was performed only for carriers, a comparison in terms of age, sex, and location was performed to assess that pig farmers and nonfarmer carrier subgroups were comparable for these variables. Frequency of co-resistance to ampicillin, streptomycin, and trimethoprim-sulfamethoxazole in predominant strains of *E. coli* was used as a marker for multiple resistance and compared between groups (*23*). In analyzing data, we did not adjust for making multiple comparisons (*24*) since adjusting remains controversial (*25*,*26*), particularly for actual observations on nature (*27*). The association between isolation of resistant strains and specific farming activities and the size of farms was assessed by chi-square analysis.

## Results

We matched 113 exposed pig farmers with 113 nonexposed nonfarmers. The overall male-to-female ratio was 6.1, and mean age was 37.8 years (range 21–72). Mean previous time in the professional position occupied at the time of the study was 9.7 +/- 1.9 and 13.0 +/- 1.6 years for pig farmers and nonfarmers, respectively (p < 0.01).

Health insurance reimbursement data showed that antimicrobial agents had been prescribed in the month preceding the study for two pig farmers (one with macrolide and one with broad-spectrum penicillin 24 and 28 days before participation, respectively) and three nonfarmers (one with oral cephalosporin, one with penicillinase-resistant penicillin, and one with tetracycline 3, 10, and 24 days before participation, respectively). However, because of the retrospective nature of this analysis, the low number of participants, the nearly even distribution between pig farmers and nonfarmers, and the fact that reimbursement data are not a formal proof that antimicrobial agents were actually taken, these five persons were included in further analysis. Neither overall, nor class-specific antimicrobial prescriptions during the 6 months preceding participation in the study were significantly different between pig farmers and nonfarmers (Table 1). Prevalence of nasal or pharyngeal isolation of *S. aureus* was significantly higher in pig farmers (PR 1.85; confidence intervals [CI] 1.26 to 2.71]; p < 0.01) (Table 2). Isolation of erythromycin-resistant strains was significantly more frequent among *S. aureus* pig farmer carriers than among nonfarmer carriers (PR 9.72; CI 2.53 to 37.30; p < 0.01). Moreover, 31 (87%) of 36 macrolide-resistant *S. aureus* isolates from pig farmers were cross-resistant to lincosamides. Five pig farmers, but no nonfarmers, had MRSA (not significant). Analysis of the antimicrobial-susceptibility profile of these strains showed that two were resistant to at least one macrolide antimicrobial agent, four were resistant to aminoglycosides, and four were resistant to pefloxacin. Three of the MRSA carriers had been hospitalized within the 2 years preceding the study, including one within the previous year. The two other farmers had not been hospitalized but had visited outpatient clinics for medical problems within the year preceding the study.

**Table 1:** Total defined daily doses (DDD) of various classes of antimicrobial agents during the 6 months preceding participation in study^a^

Antimicrobial agent	Total DDD (no. participants^b^)				
	Pig farmers			Nonfarmers		
Penicillins (narrow-spectrum, broad-spectrum, and penicillinase resistant)	138	(9)		132	(9)	
Cephalosporins	53	(7)		83	(9)	
Macrolides and lincosamides	67	(9)		35	(6)	
Others	15	(3)		67	(2)	
Total	273	(25)^b^		317	(19)^c^	

^a^As determined by health insurance reimbursements to pig farmers and nonfarmers. ^b^Who used any given type of antimicrobial agent. ^c^Some persons had multiple treatments.

**Table 2:** Nasopharyngeal isolation of *Staphylococcus aureus* with various susceptibility to antimicrobial agents in pig farmers and nonfarmers^a^

Type of *S. aureus*	Prevalence no. (%)						Prevalence ratio	CI 95%	p value	
	Pig farmers		Nonfarmers							
Any	50/112	(44.6)	27/1122		(24.1)		1.85	1.26 to 2.71	<0.01	
Resistant to											
Methicillin	5^a^/50	(10.0)	0/27					NA^c^	0.59	
Macrolides	36/50	(72.0)	2/27	(7.4)		9.72		2.53 to 37.30	<0.01	
Gentamicin	10/50	(20.0)	0/27			NA		NA	0.11	
Pefloxacin	8/50	(16.0)	1/27	(3.7)		4.32		0.57 to 32.75	0.22	

^a^Matched nasal samples were available for 112 pig farmer–nonfarmer pairs only. ^b^In addition of being resistant to methicillin, two strains were resistant to at least one macrolide antibiotic (two were resistant [R] to erythromycin, lincomycin, and pristinamycin; 1 susceptible [S] to erythromycin only; and one susceptible to pristinamycin only), 4 strains were R to aminoglycosides (2 were RRS and 2 RRR to kanamycin, tobramycin, and gentamicin, respectively). Four strains were resistant to pefloxacin. ^c^NA, not applicable.

Prevalence of pharyngeal isolation of *Streptococcus pneumoniae*, *H. influenzae,* and β-hemolytic streptococci was low and did not differ significantly between groups (Table 3). One pig farmer carried yeast (*Candida albicans)*. Isolation of nongroupable streptococci was frequent and not significantly different between groups, but that of nongroupable streptococci resistant to ampicillin was significantly more frequent in pig farmers than in nonfarmers (PR 2.02; CI 1.32 to 3.09; p < 0.01). Prevalence of fecal enterococci was not significantly different between groups nor was isolation of enterococci resistant to erythromycin or vancomycin (Table 4). In all, 16 VRE were isolated including 2 VanA-type *Enterococcus faecium*, along with 11 *E. gallinarum* and 3 *E. casseliflavus* of VanC phenotype and genotype*.* Nearly all participants carried enterobacteria: 103 (94.5%) of 109 pig farmers and 100 (91.7%) of 109 nonfarmers (PR 1.03; CI 0.96 to 1.10; not significant). Isolation of enterobacteria resistant to nalidixic acid (PR 7.12; CI 2.20 to 23.0; p < 0.01), chloramphenicol (PR 2.08; CI 1.17 to 3.68); p < 0.01), tetracycline (PR 1.65; CI 1.27 to 2.13; p < 0.01), and streptomycin (PR 1.40; CI 1.01 to 1.95; p < 0.01) was significantly more frequent in pig farmer carriers of enterobacteria than in nonfarmer carriers. Regarding the predominant flora, the most frequent species isolated were *Escherichia coli* (917/995; 92.2%) followed by *Hafnia alvei* (48/995; 4.8%) and *Citrobacter freundii* (11/995; 1.1%) with no significant between-group differences. The prevalence of isolation of *E. coli* resistant to cotrimoxazole (PR 3.02; CI 1.68 to 5.44; p < 0.01), tetracycline (PR 2.22; CI 1.48 to 3.32; p < 0.01), streptomycin (PR 1.40; CI 1.01 to 1.95; p = 0.04), or nalidixic acid (PR not calculable; p < 0.01) was significantly higher in pig farmers carrying *E. coli* than in nonfarmers (Table 4). In all instances in which subgroups of pig farmers and nonfarmers were compared, no significant between-group difference emerged in terms of age, sex, and county of residence. Prevalence of co-resistance to ampicillin, streptomycin, and cotrimoxazole was also significantly higher in *E. coli* from pig farmers (24%, 24/100) than from nonfarmers (12.2%, 12/98) (PR 1.96; CI 1.04 to 3.70; p = 0.03). No strains resistant to ceftazidime were isolated. No strains of *Clostridium difficile, Pseudomonas aeruginosa,* or *Staphylococcus aureus* were isolated from the feces of any study participant. Prevalence of yeast was not significantly different between pig farmers and nonfarmers, and the species were evenly distributed (Table 4).

**Table 3:** Pharyngeal isolation of selected microorganisms in pig farmers and nonfarmers^a,b^

Microorganisms	Prevalence, no. (%)				Prevalence ratio	CI 95%		p value
	Pig farmers		Nonfarmers					
*Streptococcus pneumoniae*	0/112	(0)	3/112	(2.7)	NA	NA		0.25
*Haemophilus influenzae*	6/112	(5.4)	5/112	(4.5)	1.20	0.38 to 3.82		1.00
*Enterobacteria*	1/112	(0.9)	2/112	(1.8)	0.50	0.05 to 5.44		1.00
Yeasts^c^	1/112	(0.9)	0/112		NA	NA		0.25
β-hemolytic streptococci^d^	11^e^/112	(9.8)	9^f^/112	(8.0)	1.22	0.53 to 2.83		0.82
NGS^g^								
Any								
Resistant to	108/112	(96.4)	100/112	(89.3)	1.08	1.00 to 1.16		0.06
Ampicillin	48/108	(44.4)	22/100	(22.0)	2.02	1.32 to 3.09		<0.01
Macrolides	108/108	(100.0)	100/100	(100.0)	NA	NA		1.00

^a^Matched pharyngeal samples were available for 112 pig farmer–nonfarmer pairs. ^b^CI, confidence interval; NA, not applicable. ^c^*Candida albicans*. ^d^Several species were present in some study participants. ^e^Group A Streptococcus:1, group C: 5, *S*. *anginosus*: 3, *S*. *intermedius*: 1, *S. constellatus*: 4. ^f^Group A Streptococcus: 1, group C: 5, *S. anginosus*: 3, *S*. *intermedius*: 1, *S. constellatus*: 3. ^g^Nongroupable streptococci.

**Table 4:** Fecal isolation of selected microorganisms in pig farmers and in nonfarmers^a^

Microorganisms	Prevalence no. (%)				Prevalence ratio	CI 95%	p value
	Pig farmers		Nonfarmers				
Enterococci							
Any	71/109	(65.1	80/109	(73.4)	0.89	0.75 to 1.05	0.21
Resistant to							
Erythromycin	38/71	(53.5)	46/80	(57.5)	0.93	0.70 to .24	0.62
Vancomycin	6^b^/71	(8.5)	10^c^/80	(12.5)	0.68	0.26 to 1.77	0.42
Enterobacteria^d^							
Any	103/109	(94.5)	100/109	(91.7)	1.03	0.96 to 1.10	0.58
Resistant to							
Ampicillin	68/103	(66.0)	55/100	(55.0)	1.20	0.96 to 1.50	0.11
Ceftazidime	0		0		NA^d^	NA	NA
Streptomycin	69/103	(67.0)	48/100	(48.0)	1.40	1.09 to 1.78	<0.01
Kanamycin	29/103	(28.2)	23/100	(23.0)	1.22	0.76 to 1.96	0.40
Gentamicin	10/103	(9.7)	3/100	(3.0)	3.24	0.92 to 11.42	0.05
Chloramphenicol	30/103	(29.1)	14/100	(14.0)	2.08	1.17 to 3.68	<0.01
Tetracycline	73/103	(70.9)	43/100	(43.0)	1.65	1.27 to 2.13	<0.01
Nalidixic acid	22/103	(21.4)	3/100	(3.0)	7.12	2.20 to 23.0	<0.01
*Escherichia coli* ^f^							
Any	100/109	(91.7)	98/109	(89.9)	1.02	0.94 to 1.10	0.64
Resistant to							
Ampicillin	36/100	(36.0)	34/98	(34.7)	1.04	0.71 to 1.51	0.85
Ceftazidime	0		0		NA	NA	NA
Streptomycin	50/100	(50.0)	35/98	(35.7)	1.40	1.01 to 1.95	0.04
Kanamycin	10/100	(10.0)	12/98	(12.2)	0.82	0.37 to 1.80	0.62
Gentamicin	2/100	(2.0)	0		NA	NA	0.99
Chloramphenicol	11/100	(11.0)	9/98	(9.2)	1.20	0.52 to 2.76	0.67
Tetracycline	52/100	(52.0)	23/98	(23.5)	2.22	1.48 to 3.32	<0.01
Cotrimoxazole	37/100	(37.0)	12/98	(12.2)	3.02	1.68 to 5.44	<0.01
Nalidixic acid	11/100	(11.0)	0		NA	NA	<0.01
*Staphylococcus aureus*	4/109	(3.7)	2/109	(1.8)	2.0	0.37 to 10.69	0.68
Yeasts	19^g^/109	(17.4)	18^h^/109	(17.4)	1.06	0.59 to 1.90	1.00

^a^Matched fecal samples were available for 109 pig farmers and nonfarmer pairs only. ^b^*Enterococcus faecium*: 0, *E. gallinarum*: 6. ^c^*E. faecium*: 2, *E. gallinarum*: 5, *E. casseliflavus*: 3. ^d^Using direct plating plating on Drigalski agar without or with antimicrobial agents (first technique, see Methods). ^e^NA, not applicable. ^f^From the predominant fecal flora (second technique, see Methods). ^g^*Candida albicans*: 1, *Geotrichum* sp.: 15, *C. glabrata*: 2, *Rhodolulora* sp.: 1. ^h^*C. albicans*: 2, *Geotrichum* sp.: 14, *Saccharomyces cerevesia*: 2.

Most pig farmers had several professional activities. Only a few farmers used isolation precautions (Table 5). We found no statistical association between professional activity or use of masks and gloves and the prevalence of resistant bacteria. By contrast, prevalence of nasal isolation of *S. aureus* resistant to macrolides increased significantly, from 33% (5/15) in pig farmers working in farms raising 84–180 swine, to 70% (7/10), 92% (11/12), and 100% (13/13) in those working in farms raising 181–270, 271–399, and >400 swine, respectively (chi-square linear slope; p < 0.01).

**Table 5:** Frequency of use of masks and gloves by 113 pig farmers during selected farming activity

Activity	No. (%) with that activity	No. (%) using^a^	
		Masks	Gloves
Food preparation, daily or often	109 (96)	4 (3.6)	8 (7.3)
Manual food distribution, handling, or mixing	78 (69)	4 (5.2)	5 (6.4)
Handling of pig feces, daily or often	87 (77)	2 (2.3)	7 (8.0)
Antibiotic administration to animals	112 (99)	4 (3.5)	9 (8.0)

^a^During that activity.

## Discussion

Our results showed that the prevalence of antimicrobial drug resistance in bacteria from the nasal, pharyngeal, and fecal flora was higher in pig farmers than in nonfarmers. With a few exceptions, pig farmers and nonfarmers had not taken antimicrobial agents during the month preceding the study and had not been differentially exposed to such agents during the previous 6 months. That *E. coli* (*11*–*13*) and enterococci (*14*) are significantly more resistant in persons working in farms or slaughterhouses than in urban residents had been reported, but a potential role of antimicrobial treatments in these workers could not be excluded and the increased prevalence of carriage of resistant organisms had not been quantified.

The prevalence of *S. aureus* nasal carriage in nonfarmers was similar to that reported previously in the general population (*28*), which suggests that the higher isolation rate in pig farmers was due to their work environment. This hypothesis was further supported by the increased resistance to macrolides (still the fourth most common class of antimicrobial agents used in food production [*8*]) of *S. aureus* isolates from pig farmers and the link between this resistance and the size of the farm. Why the isolation rate of *S. aureus* was higher in pig farmers remains unclear. Several hypotheses, including high transfer of animal specific clones, should be raised and investigated.

In the pharynx, ampicillin resistance of nongroupable streptococci in pig farmers may contribute to further transfer of β-lactam resistance to *Streptococcus pneumoniae* by transformation (*29*). In the feces, antimicrobial drug resistance in enterobacteria was also greater in pig farmers for four of eight markers tested in the subdominant flora, and for four of nine markers in the predominant flora. Resistance in *E. coli* was close to that of healthy participants from developing countries (*22*). The prevalence of resistance in enterobacteria from the subdominant flora of our nonfarmers was lower than that in participants of the only study published that used the same methods; however, that study included mostly laboratory workers (A. Andremont, pers. comm.), who are known to be more colonized by resistant enterobacteria than are urban and rural dwellers (*30*). The rate of VRE colonization that we observed differed from that reported in France (*31*), which might be due to the enrichment step we used; however, the rate of VRE colonization did not differ between farmers and nonfarmers. This finding suggests that the 1997 ban (*32*) of avoparcin, a glycopeptide previously used as a growth promoter, was effective. Although specific information on avoparcin is lacking, 145 tons of antimicrobial agents were used globally in France in 1998 in pig raising, including 70 mg of growth additive per kilogram of pork meat produced (*33*).

Three possible explanations may explain why isolation of resistant bacteria in pig farmers was higher than in nonfarmers. First, farmers may come in contact with more antimicrobial-resistant bacteria from pigs; these bacteria are then transferred to the farmers. Second, farmers may be in frequent contact with antimicrobial agents themselves or antimicrobial residues that are given to the pigs in the workplace. The third possibility is that farmers receive more antimicrobial agents for other, i.e., medical, reasons. The first of these possibilities appears most likely because 1) farmers used very few precautions during contact with animal feces, 2) antimicrobial exposure is a well-known risk factor for intestinal yeast colonization (*34*,*35*), and yeast colonization in both groups was low, and 3) antimicrobial prescriptions were not significantly different between pig farmers and nonfarmers during the previous 6 months.

We did not assess the use of antimicrobial agents for animals in each of the 113 farms where pig farmers worked. However, 1,364 tons of antimicrobial agents were sold in France in 1999 for veterinary medicinal use. Of these, tetracycline, cotrimoxazole, and β-lactams together accounted for 79.5% (*8*), a finding compatible with the high resistance rates found in pig farmers. However, we could not assess the exact cause of the high antimicrobial resistance rates in farmers. Determining the exact cause may not be as important as the fact that these people are colonized with a much higher rate of resistant bacteria. Further studies will need to be undertaken to identify the cause of this phenomenon.

Food products are a source of resistant bacteria (*9*,*10*). We minimized the risk that differences in food intake caused the higher prevalence of resistance in pig farmers by matching pig farmers with nonfarmers by age, sex, and county of residence. Children can be a source of resistant bacteria in households (*36*) and thus might be a confounding factor if the number of children was greater in pig farmer families than in nonfarmer families. However, this factor was not documented in the study questionnaire and thus could not be investigated.

Some inherent limitations of cross-sectional studies invite cautious assessments of our results. The lack of preexposure data on resistance and the general design of the study preclude determining a causal relationship between exposure and acquired resistance. However, the observation we made indicates that professional pig farming is significantly associated with isolation of antimicrobial-resistant commensal species. The minimal use of contact precautions by pig farmers may have further increased this risk, but the study was not designed to assess the efficacy of contact precautions, and thus no recommendations can be drawn in this matter.

Pigs could be raised with considerably fewer antimicrobial agents than currently used, and many animals can be raised with little or no exposure to such drugs at all (*37*). However, antimicrobial agents will still be used to treat sick animals. Additional studies are needed to evaluate the consequences of isolating resistant bacteria in farmers and, if necessary, design appropriate preventive measures.
